# Study on the Mechanism of Reducing Hepatotoxicity of Water-Grinding Realgar by Metabolomics, Morphology, and Chemical Analysis

**DOI:** 10.1155/2021/8538287

**Published:** 2021-12-14

**Authors:** Ting Han, Hui Zhang, Wenjuan Xu, Chunshuai Li, Min Wang, Yuying Bai, Linlin Yang, Shuyan Zhang, Zhe Jia, Xinfang Xu, Chongjun Zhao, Feng Wei, Xiangri Li

**Affiliations:** ^1^Beijing Key Laboratory for Quality Evaluation of Chinese Materia Medica, School of Chinese Materia Medica, Beijing University of Chinese Medicine, Beijing, China; ^2^Merck Sharp & Dohme Ltd., Beijing, China; ^3^National Institutes for Food and Drug Control, Beijing, China

## Abstract

**Background:**

Realgar was usually selected as a substitute for arsenic trioxide to treat acute promyelocytic leukemia due to its higher effect without high cardiotoxicity. In traditional Chinese medicine (TCM), realgar is usually processed by the water-grinding method clinically, but the mechanism of realgar processing detoxification is still unclear. However, it is necessary to take safety and efficacy into account while evaluating a drug.

**Methods:**

Sixty male Wistar rats were divided into control group, realgar products-treated groups, and corresponding subgroups. Biochemistry analysis and histopathological examination were performed in the study, and plasma samples were collected from all the rats for metabolomics analysis.

**Results:**

No significant toxicity was observed in rats treated with 0.64 g/kg/day grinding realgar (G-r) and water-grinding realgar (WG-r). When the dose increased to 1.92 g/kg/day, the liver weight coefficients of the rats treated with G-r (HG-r: 3.65 ± 0.26%) and WG-r (HWG-r: 3.67 ± 0.14%) increased significantly and severe hepatic injury occurred in comparison to the control group (Group C: 3.00 ± 0.21%). After one week's withdrawal, the liver injury caused by the high dose of WG-r significantly recovered, while the liver damage caused by G-r was more difficult to recover. In metabolomics analysis, 14 metabolites were identified as the potential biomarkers in realgar-treated rats. These metabolites indicated that there were perturbations of the primary bile acid biosynthesis, arachidonic acid metabolism, linoleic acid metabolism, and glycerophospholipid metabolism in the realgar-treated groups.

**Conclusions:**

These results illustrate that, as a TCM processing method, water grinding had the effect of reducing toxicity, and the metabolomics method may be a valuable tool for studying the toxicity induced by TCM and the mechanism of TCM processing.

## 1. Introduction

Arsenic is known to be poisonous, and it has recently attracted attention as a therapy for cancer [1]. Arsenic trioxide (ATO, As_2_O_3_) is a characteristic example applied to treat acute promyelocytic leukemia (APL) successfully in clinic [2]. However, the use of ATO has limitations for its dose-dependent side effects including cardiotoxicity [3], hematological toxicity [4], neurotoxicity [5], and hepatotoxicity [6], which deterred several physicians from applying it to APL patients in clinic. Therefore, other arsenic derivatives with similar efficacy but less toxicity would be desirable.

Realgar, containing more than 90% tetra-arsenic tetra-sulfide (As_4_S_4_), is called Xionghuang in traditional Chinese medicine (TCM) and usually contains trace ingredients, of which As_2_O_3_ is the main component. The clinical application of realgar has a long history. It was first recorded in the 2000-year-old classic, Shen Nong Ben Cao Jing. It was used to treat carbuncle, bites caused by insects or snakes, convulsions, intestinal parasitosis, and epilepsy for thousands of years [7]. In addition, Chinese patent medicines containing realgar, such as Angong Niuhuang pills and Niuhuang Jiedu tablets, were widely used in clinic. Compared with ATO, realgar is considered to have less adverse effect because of its high lattice energy and low free arsenic concentration, and thus it was a substitute with a good reputation in TCM [8]. Besides, realgar caused degeneration and apoptosis of APL promyelocytes in morphology rather than differentiation [9]. But meanwhile, evaluating the safety and efficacy of the drug should be taken into account.

The water-grinding processing method, a commonly used method in traditional clinical mineral medicine, is an effective way to reduce soluble impurities, increase the dissolution rate, and enhance the bioavailability of realgar without affecting its curative effect [10]. Although water-grinding realgar (WG-r) only has trace amounts of soluble arsenic, the element “As” is still a potential risk in WG-r. Previous research works have studied the hepatotoxicity of realgar in rats by LC-MS, GC-MS, or ^1^H NMR, but they did not determine whether the process of water grinding can alleviate the hepatotoxicity of realgar. Metabolomics is an emerging “omics” technology focusing on exploring metabolites changes in microorganisms, plants, and animals [11, 12]. The metabolomics quantitative analysis based on mass spectrometry has high selectivity, and it can be used for identifying potential metabolites. In the past two decades, mass spectrometry-based metabolomics, cooperating with other systems biology approaches, has been used to improve our comprehending of disease state, drug effect, and toxicity [13, 14]. In this study, histopathology, biochemical assay, and plasma metabolomics were used in conjunction with multivariate statistical analysis to study the hepatotoxicity of WG-r and grinded realgar (G-r) of the same particle size in rats for a long time. Additionally, this study also aimed to elucidate the potential toxicity mechanism and to probe that whether the processing by water can reduce the hepatotoxicity of realgar. By comparing the hepatotoxicity between WG-r and G-r and observing whether it can recover after drug withdrawal, we may be able to explain professionally the clinical use of realgar after water grinding.

## 2. Materials and Methods

### 2.1. Chemicals and Regents

Realgar was obtained from Guizhou Province, China. It was identified as the sulfide mineral realgar by Yaojun Yang, a professor of the Pharmacognosy Department at the Beijing University of Chinese Medicine. Voucher specimens (XH 1508) were deposited at the School of Chinese Materia Medica, Beijing University of Chinese Medicine.

### 2.2. Processed Material Preparations and Their Characterization

The water-grinding realgar product (WG-r) was processed with water according to the Chinese Pharmacopoeia (2020 edition), and the grinding realgar (G-r) was ground without water to the same level as the diameter of WG-r, and the particle size was detected using a BT-9300Z laser particle size analyzer (Bettersize Instruments Ltd., China). The detailed characteristics of G-r and WG-r were confirmed using a Quanta 250FEG scanning electron microscope (FEI Company, US). The content of As_4_S_4_ in G-r (98.66%) and WG-r (98.56%) were detected by the oxidation reduction titration method. The soluble arsenic content in G-r (2.47%) and WG-r (2.79%) were analyzed using an AA-6880F atomic absorption spectrophotometer (Shimadzu, Japan). Voucher specimens were deposited at the School of Chinese Materia Medica, Beijing University of Chinese Medicine.

The WG-r and G-r powders were suspended with 0.5% (w/v) CMC-Na.

### 2.3. Animals and Treatment

Animal care was conducted in accordance with the ethical guidelines for the Purpose of Control and Supervision of Experiments on Rats approved by the Animal Ethics Committee of the Beijing University of Chinese Medicine. All efforts were made to minimize the number and suffering of the animals.

Sixty male Wistar rats, 180 ± 20 g, were obtained from SPF-Biotechnology Co., Ltd., Beijing, China, and fed with commercial food purchased from Beijing Keao Xieli Feed Co., Ltd. All rats were housed at 20–23 °C with a relative humidity of 40%–60%, under artificial lighting (12-h light-dark cycle). After acclimatization for seven days, rats were randomly assigned to the control group (*n* = 12), the G-r group (*n* = 24), and the WG-r group (*n* = 24). In order to investigate the effects of different doses of realgar and realgar withdrawal on the liver, after being treated with 0.5% (w/v) CMC-Na or regular products for a successive month, these rats were further randomly divided into treatment subgroups and recovery subgroups.  Table 1 shows the information of groups.

The dosages of realgar 1.92 g/kg/day bodyweight and 0.64 g/kg/day bodyweight were set according to our previous study. The rats were weighed every 3 days, and the dosages of G-r and WG-r were adjusted for the bodyweight of each rat. The clinical reactions of rats in ten groups were observed within 4 hours after oral administration. After the last lavage, the rats were sacrificed by only giving water without any food for 24 h.

### 2.4. Sample Collection

Rats in the control group (Group C), low-grinding-realgar group (Group LG-r), high-grinding-realgar group (Group HG-r), low-water-grinding-realgar group (Group LWG-r), and high-water-grinding-realgar group (Group HWG-r) were sacrificed on the 30th day, and rats in the convalescent control group (Group CC), low-grinding-realgar convalescent group (Group CLG-r), high-grinding-realgar convalescent group (Group CHG-r), low-water-grinding-realgar convalescent group (Group CLWG-r), and high-water-grinding-realgar convalescent group (Group CHWG-r) were sacrificed on the 37th day.

The plasma samples were centrifuged at 3500 rpm for 10 min at 4°C from the blood samples taken from the eyeball vein plexus of rats and stored in a refrigerator at −80°C until UPLC-HDMS analysis. The serum samples were centrifuged at 3500 rpm for 15 min at 4°C from the blood samples taken from the abdominal aorta of rats and stored in a refrigerator at −80°C until biochemical assay. The livers were immediately excised from the rats, weighed accurately, and divided into two aliquots. One aliquot was fixed in 10% formalin solution for subsequent histopathological analysis, and the other was stored at −80°C until homogenized for biochemical assay.

### 2.5. Biochemical Assay

The contents of aspartate aminotransferase (AST) and alanine aminotransferase (ALT) in the serum were detected using an automatic hematology analyzer (Beckman Coulter, Inc.) to evaluate the hepatotoxicity. The contents of antioxidant index superoxide dismutase (SOD) and glutathione (GSH) and oxidative damage index malondialdehyde (MDA) in the liver homogenate were tested using assay kits (Nanjing Jiancheng Bioengineering Institute, China). All operations are carried out in accordance with the instructions strictly.

### 2.6. Histopathology

The liver fixed in formalin was embedded in paraffin and cut into slices. The sections were stained with H&E for the following histological analysis. Representative photomicrographs were taken to investigate ultrastructural changes under a light microscope.

### 2.7. Metabolomics Analysis

A 200 *μ*L aliquot of plasma was mixed with 600 *μ*L of acetonitrile. The mixture was vortexed for 2 minutes to precipitate protein in the plasma. After centrifugation (14,000 rpm, 10 min, 4°C), 600 *μ*L of supernatant was pipetted out and dried with nitrogen. Then, it was dissolved in 100 *μ*L of water:acetonitrile (20 : 80) and vortex-mixed for 2 min. The supernatant was drawn for UPLC-HDMS after centrifuging at 14,000 rpm for 10 min at 4°C.

Quality control (QC) samples, a mixture pooled by an equal volume of all plasma from six groups, were prepared by the same method as the sample preparation described above. Six QC samples were prepared in this study. To make sure the UPLC-HDMS method satisfied with the requirements of metabolomics, the precision of the instrument and the method reproducibility were validated by 6 injections from the same QC sample and 6 injections from the different QC samples, respectively. In the whole sequence, the QC sample was injected into the instrument once for every 10 samples, which was used to evaluate system stability.

### 2.8. Apparatus and Analytical Conditions

Chromatographic separation was performed on a 2.1 × 100 mm, 1.6 *μ*m C_18_ column (Waters, Ireland) with an Accela 600 pump LC system (Thermo Scientific, Bremen, Germany) equipped with a binary pump and an autosampler. The UPLC mobile phase consisted of water with 0.1% formic acid (solution A) and acetonitrile (solution B). The flow rate was 0.30 mL/min, and the linear gradient elution program was as follows: 0–3.0 min, 5–40% B; 3.0–10.0 min, 60–70% B; 10.0–20.0 min, 70–80% B; 20.0–25.0 min, 80–95% B; 25.0–30.0 min: 95–5% B. The analytical column and autosampler were maintained at 40°C and 4°C, and 1 *μ*L aliquot of each sample was injected for analysis.

Mass spectrometry was performed on an LTQ Orbitrap mass spectrometer (Thermo Scientific, Bremen, Germany) connected to the UPLC instrument via an ESI interface. Samples were analyzed in both negative mode and positive mode with the tune method set as follows: sheath gas (nitrogen) flow rate of 40 arb, aux gas (nitrogen) flow rate of 20 arb, source voltage of 4.0 kV, capillary temperature of 350°C, capillary voltage of 25 V, tube lens voltage of 110 V. The measured masses were within 5 ppm of the theoretical masses. The scan range was from 100 to 1500 m/z.

### 2.9. Data Analysis

The raw UPLC-MS data of plasma samples were imported into XCMS online software and then processed with default settings to complete baseline correction, peak discrimination and alignment, and retention time correction. The result matrix was normalized to the sum of all peak areas of each sample and then handled by “80% rule” to eliminate the missing value. The relative standard deviation (RSD) was applied to validate the instrumental precision, method reproducibility, and system stability. In this study, after alignment and normalization, data with RSD higher than 30% in QC samples were removed.

The unsupervised PCA and the supervised OPLS-DA were performed on SIMCA-P 13.0 (Umetrics, Umea, Sweden). The potential metabolite markers were selected by variable importance (VIP) values in the OPLS-DA model greater than 1.00 and Student's *t*-test *P* values less than 0.05. The data from clinical biochemistry were expressed as the means ± standard deviations, and the statistical analysis was performed using SPSS 17.0 (IBM, New York, USA). A statistically significant difference was accepted when *P* < 0.05.

Identification of potential biomarkers were based on the MS/MS fragment ion and matched with the structure message from HMDB (http://www.hmdb.ca/) [15] and METLIN (http://metlin.scripps.edu/). The pathway analysis and correlation analysis of potential biomarkers were performed by MetaboAnalyst 4.0 (http://www.metaboanalyst.ca/) [16] and KEGG (https://www.kegg.jp). The Pearson correlation coefficient was calculated by a cor() function and visualized by using the corrplot package.

## 3. Results

### 3.1. Particle Size and Morphology of G-r and WG-r

**Figure 1:**
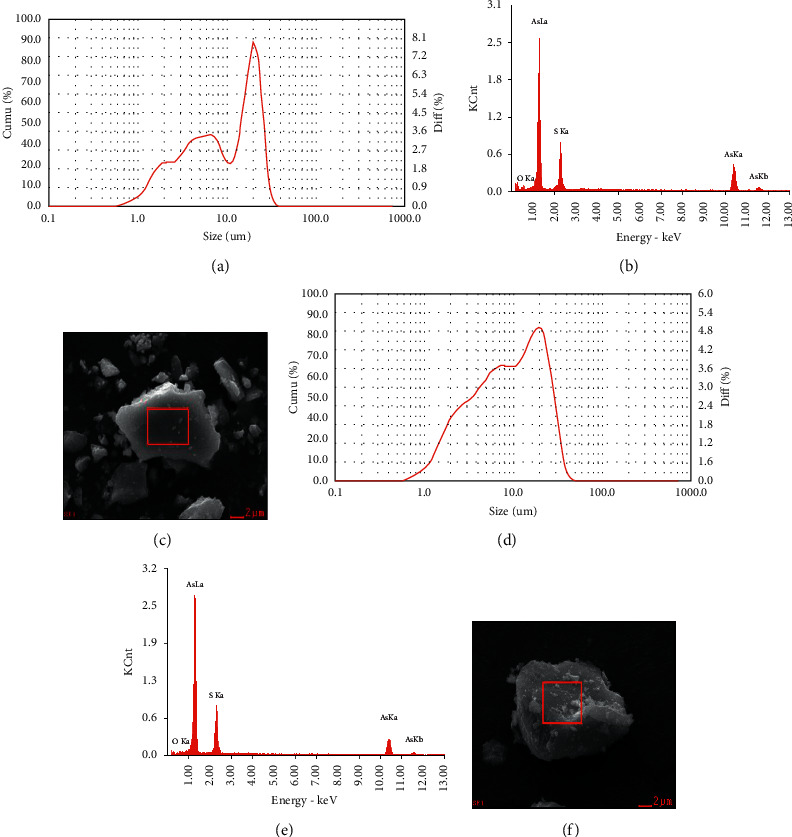
Particle sizes and morphologies of G-r and WG-r. (a, d) Particle size analysis reports of G-r (a) and WG-r (d). (b, e) Energy spectrum diagrams of G-r (b) and WG-r (e). (c, f) SEM secondary electronic images of morphology of G-r (c) and WG-r (f).

The particle sizes of G-r and WG-r were at the same level (median: 8.985 µm vs. 8.983 µm). Oxygen, sulfur, and arsenic elements were all detected in G-r and WG-r by elemental analysis (Figures 1(b) and 1(e)). Scanning electron microscopy (SEM) showed that G-r and WG-r have different morphologies. The surface texture of WG-r is clear and smooth, obtuse, and round, with more fine powders attached to the surface. G-r, on the other hand, had more polyhedral with sharp edges (Figures 1(c) and 1(f)). The total arsenic content of G-r and WG-r was 98.66% and 98.56%, and the soluble arsenic content was 2.47% and 2.79%, respectively.

### 3.2. General Observations and Weight Changes

Compared with the control group (Group C), the rats' feces in the drug-treated groups (Group LG-r, HG-r, LWG-r, and HWG-r) were orange, and the rats' feces in the convalescent groups (Group CLG-r, Group CHG-r, Group CLWG-r, and CHWG-r) returned to normal without medicine for seven days. Changes of whole bodyweight from the first day to last day had no significant difference between each group (*P* > 0.05, the data were not shown in this study), which indicated that different doses of realgar products had no effect on the normal growth of rats in the study.

### 3.3. Histopathology

**Figure 2:**
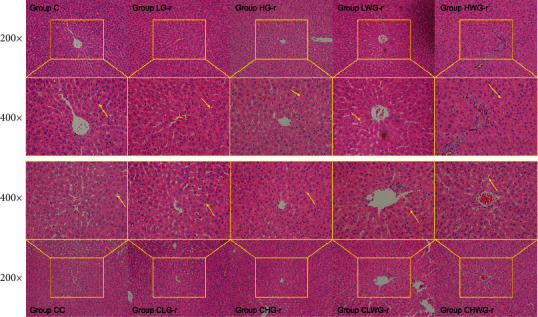
Photomicrographs of representative liver sections of rats exposed to different realgar products. Group C, control group; Group LG-r, low-dose grinding-realgar group; Group HG-r, high-dose grinding-realgar group; Group LWG-r, low-dose water-grinding-realgar group; Group HWG-r, high-dose water-grinding-realgar group; Group CC, convalescent control group; Group CLG-r, low-dose grinding-realgar convalescent group; Group CHG-r, high-dose grinding-realgar convalescent group; Group CLWG-r, low-dose water-grinding-realgar convalescent group; Group CHWG-r, high-dose water-grinding-realgar convalescent group.

Histopathological results expressed that the liver section of rats had no signs of abnormality observed in Group C and Group CC (Figure 2). Compared with those two groups, cloudy swelling, cell cord derangement of hepatocytes, and hepatic sinusoid stenosis were observed in rats exposed to the high-dose groups of G-r (Group HG-r) and WG-r (Group HWG-r; Figure 2), but this pathological phenomenon was not obvious in the low-dose groups (Group LG-r and Group LWG-r). Rats of Group CHWG-r showed recovery trends for liver injuries induced by high-dose WG-r, which was not obvious in Group CHG-r.

### 3.4. Liver Weight Coefficient and Biochemical Results

**Figure 3:**
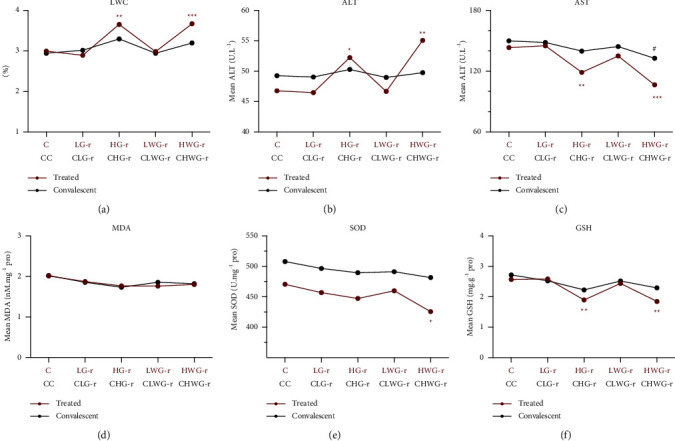
Liver weight coefficient and biochemical analyses. The data are presented as means ± SD. LWC: liver weight coefficients, ALT: alanine aminotransferase, AST: aspartate aminotransferase, SOD: superoxide dismutase, MDA: malondialdehyde, GSH: glutathione. ^*∗*^*P* < 0.05, ^*∗∗*^*P* < 0.01, ^*∗∗∗*^*P* < 0.01, significant difference compared with Group C. #*P* < 0.05, significant difference compared with Group CC. The Welch ANOVA followed by Tukey's multiple comparison test or Student–Newman–Keuls (SNK) test was used for statistical analysis. *P* < 0.05 was considered statistical significance.

Compared with the control group, liver weight coefficients (LWC, LWC = liver weight in grams/body weight in grams × 100%) of Group HG-r (3.65 ± 0.26% vs. 3.00 ± 0.21%, *P* < 0.01) and Group HWG-r (3.67 ± 0.14%. vs. 3.00 ± 0.21%, *P* < 0.001) increased significantly (Figure 3(a)). There was no significant difference among Group CHG-r, Group CHWG-r, and Group CC (Figure 3(a)).

Compared with the control group, Group LWG-r and Group LG-r had no significant effect on the levels of ALT and AST in rats. When the dose increased to 1.92 g/kg/day, the levels of ALT in Group HWG-r (55.07 ± 2.96 U/L vs. 46.77 ± 4.37 U/L, *P* < 0.01) and Group HG-r (52.23 ± 2.83 U/L vs. 46.77 ± 4.37 U/L, *P* < 0.05) increased significantly (Figure 3(b)), and the levels of AST in Group HG-r (118.88 ± 12.10 U/L vs. 143.33 ± 12.65 U/L, *P* < 0.01) and Group HWG-r (106.52 ± 11.95 U/L vs. 143.3 ± 12.65 U/L, *P* < 0.001) decreased significantly (Figure 3(c)). However, after drug withdrawal, the levels of ALT and AST in those two groups recovered spontaneously (Figures 3(b) and 3(c)).

Biochemical parameters studies have shown that the liver homogenate SOD (*P* < 0.05) content in Group HWG-r (425.59 ± 20.34 U/mg prot) was evidently lower than Group C (470.65 ± 39.22 U/mg prot) (Figure 3(e)). Compared with Group C (2.57 ± 0.35 mg/g prot), there was a significant decrease in the levels of GSH in Group HG-r (1.90 ± 0.37 mg/g prot, *P* < 0.01) and Group HWG-r (1.84 ± 0.34 mg/g prot, *P* < 0.01) (Figure 3(f)). These biochemical indexes with significant changes basically returned to normal after discontinuation of administration (Figures 3(e) and 3(f)).

### 3.5. Multivariate Statistical Analysis

Based on UPLC-HDMS analysis, the representative based peak intensity (BPI) chromatograms of plasma from the control and treated groups were collected in positive and negative modes, respectively. The subtle metabolism variations among these complex data could be required using multivariate statistical analysis techniques, such as PCA and OPLS-DA.

**Figure 4:**
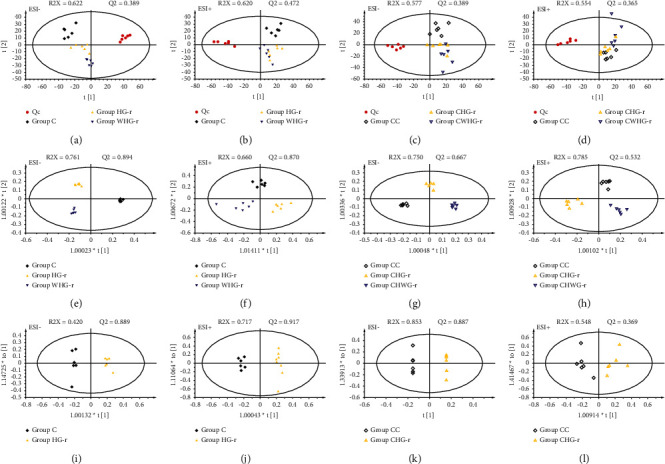
PCA and OPLS-DA plots with different groups. Scores plots of principal components analysis and orthogonal partial least squares discriminant analysis models with the statistical parameters as follows: (a) R2X = 0.622, *Q*2 = 0.389; (b) R2X = 0.620, *Q*2 = 0.472; (c) R2X = 0.577, *Q*2 = 0.389; (d) R2X = 0.554, *Q*2 = 0.365; (e) R2X = 0.761, *Q*2 = 0.894; (f) R2X = 0.660, *Q*2 = 0.870; (g) R2X = 0.750, *Q*2 = 0.667; (h) R2X = 0.785, *Q*2 = 0.532; (i) R2X = 0.420, *Q*2 = 0.889; (j) R2X = 0.717, *Q*2 = 0.917; (k) R2X = 0.853, *Q*2 = 0.887; (l) R2X = 0.548, *Q*2 = 0.369. QC, quality control group; Group C, control group; Group HG-r, high-dose grinding-realgar group; Group HWG-r, high-dose water-grinding-realgar group; Group CC, convalescent control group; Group CHG-r, high-dose grinding-realgar convalescent group; Group CHWG-r, high-dose water-grinding-realgar convalescent group.

Histopathological and biochemical results showed that high-dose realgar products had hepatotoxic effect, whereas low-dose products had little hepatotoxic effect. Metabolomics was used to analyze the hepatotoxicity caused by high-dose realgar products. At the first step of metabolomics analysis, PCA was often operated to visualize outliers, clustering, or trends in the observations. In Figures 4(a) and 4(b), the PCA score plot showed that the plasma samples among QC, Group C, Group HG-r, and Group HWG-r were obviously separated in positive and negative modes. The results indicated that control and high-dose drug-treated groups had different metabolic profiles resulting from the variance of metabolites between these three groups. QC samples were clustered tightly, which indicated the analyses have a good system.

As for three groups with recovery periods (Group CC, Group CHG-r, and Group CHWG-r), we found that the trend of differences among high-dose drug-treated convalescent groups (Group CHG-r and Group CHWG-r) and convalescent control group (Group CC) were declined (Figures 4(c) and 4(d)).

To increase the separation and discover the endogenous metabolites related to hepatotoxicity, a supervised OPLS-DA was employed in this study. The results of OPLS-DA (Figures 4(e) and 4(f)) showed an appreciable separation among Group C, Group HG-r, and Group HWG-r. Combined with variable importance for projection (VIP) values larger than 1.00 and *P* value less than 0.05, 181 metabolite variables at negative ESI mode and 115 metabolite variables at positive ESI mode were found for further identification between Group C and Group HG-r.

### 3.6. Identification of Biomarkers and Biochemical Interpretation

Subsequent feature identification was counted on metabolite databases, such as METLIN (https://metlin.scripps.edu), HMDB (https://hmdb.ca), and KEGG (https://www.kegg.jp). For improving analytic accuracy, the molecular weight tolerance was set to ±10 ppm. Compared with the standard references, the MS/MS spectrum in online databases and literatures, six metabolites at positive ESI mode and eight metabolites at negative ESI mode were tentatively identified (Table 2).

**Figure 5:**
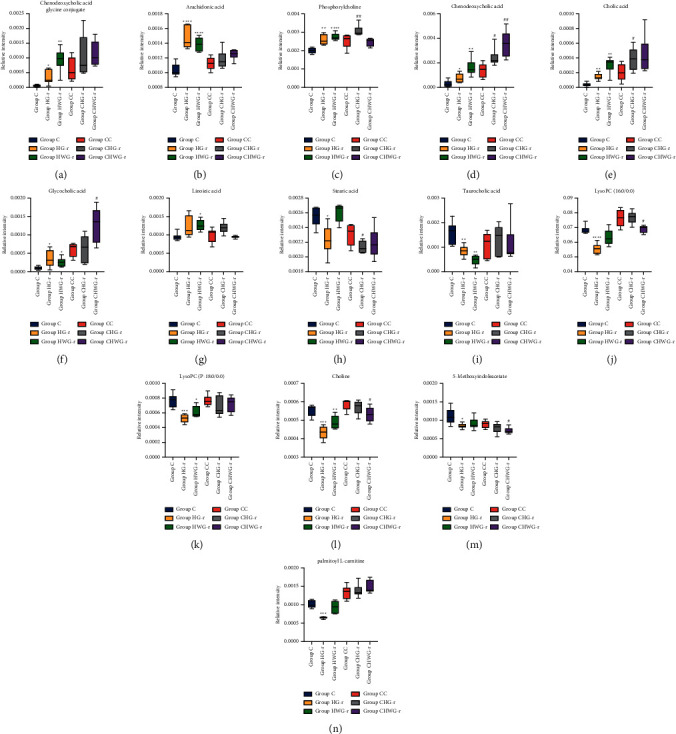
Box plots of biomarkers from plasma samples of six groups. The data are presented as means ± SD. (a) Chenodeoxycholic acid glycine conjugate. (b) Arachidonic acid. (c) Phosphorylcholine. (d) Chenodeoxycholic acid. (e) Cholic acid. (f) Glycocholic acid. (g) Linoleic acid. (h) Stearic acid. (i) Taurocholic acid. (j) LysoPC (16 : 0/0 : 0). (k) LysoPC (P-18 : 0/0 : 0). (l) Choline. (m) 5-Methoxyindoleacetate. (n) Palmitoyl L-carnitine. ^*∗*^*P* < 0.05, ^*∗∗*^*P* < 0.01, ^*∗∗∗*^*P* < 0.001, ^*∗∗∗∗*^*P* < 0.0001, significant difference compared with control group (Group C). #*P* < 0.05, ##*P* < 0.01, significant difference compared with Group CC. The Welch ANOVA followed by Tukey's multiple comparison test or Student–Newman–Keuls (SNK) test was used for statistical analysis. *P* < 0.05 was considered statistical significance.

After exposed to the high dose of G-r and WG-r, chenodeoxycholic acid glycine conjugate, arachidonic acid, phosphorylcholine, chenodeoxycholic acid, cholic acid, glycocholic acid, and linoleic acid were significantly increased in plasma samples (Figures 5(a)–5(g)). Moreover, compared with Group C, a significant decrease was observed in Group HG-r and Group HWG-r in the levels of stearic acid, taurocholic acid, Lyso phosphatidylcholines (LysoPC), choline, 5-methoxyindoleacetate, and palmitoyl L-carnitine (Figures 5(h)–5(n)), and these metabolic disorders were more severe in Group HG-r. After a one-week recovery period, except phosphorylcholine and chenodeoxycholic acid, the other 12 metabolites in Group CHG-r and Group CHWG-r were comparable to that in Group CC (Figure 5).

### 3.7. Clustering and Correlation Analysis

**Figure 6:**
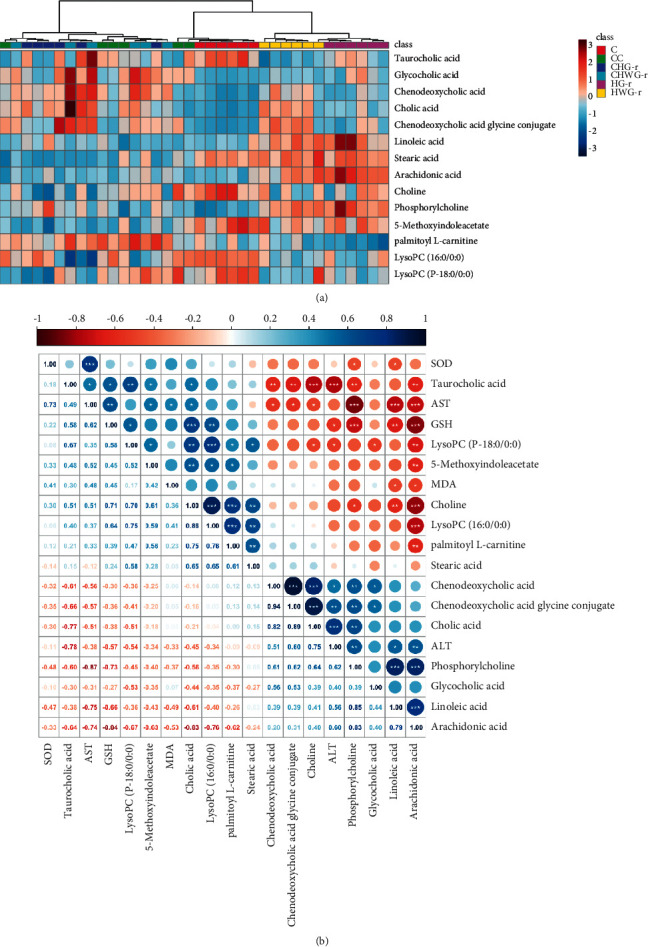
Clustering analysis and Pearson's correlations of potential biomarkers and biochemical indicators. (a) Heat map for biomarkers appeared in high-dose drug-treated groups. The color of each section is proportional to the significance of change of metabolites (blue, upregulated; red, downregulated). (b) Correlation analysis between biochemical parameters and potential biomarkers. ^*∗*^*P* < 0.05, ^*∗∗*^*P* < 0.01, ^*∗∗∗*^*P* < 0.001.

To visualize the differences among the control group, drug-treated groups, and their recovery subgroups, a heat map colored according to the relative abundances of endogenous metabolites was established in this study (Figure 6(a)). According to the clustering of heat map, there are obvious differences among Group C, Group HG-r, and Group HWG-r, with small differences among Group CC, Group CHG-r, and Group CHWG-r.

Pearson's correlation matrix analysis was used to analyze the correlation between biochemical parameters and potential biomarkers (Figure 6(b)). As shown in Figure 6(b), it has different ranges of correlation coefficients among biochemical parameters and potential biomarkers from −1.0 (maximum negative correlation) to 1.0 (maximum positive correlation), with 0 indicating no correlation. GSH was significantly negative associated with linoleic acid (*r* = −0.66, *P*< 0.01), arachidonic acid (*r* = −0.84, *P* < 0.001), and phosphorylcholine (*r* = −0.73, *P* < 0.001) and significantly positive associated with taurocholic acid (*r* = 0.58, *P* < 0.05) and LysoPC (*r* = 0.64, *P* < 0.001). No significant associations between MDA or GSH and metabolites were observed. However, positive and negative correlations between potential biomarkers and ALT and AST activities were also observed. ALT was significantly positive associated with cholic acid (*r* = 0.75, *P* < 0.001), chenodeoxycholic acid glycine conjugate (*r* = 0.60, *P* < 0.01), linoleic acid (*r* = 0.56, *P* < 0.05), arachidonic acid (*r* = 0.59, *P* < 0.01), and phosphorylcholine (*r* = 0.62, *P* < 0.01) and significantly negative associated with taurocholic acid (*r* = −0.78, *P* < 0.001). Interestingly, these correlations between AST and metabolites are completely opposite to that of ALT. AST was significantly negative associated with cholic acid (*r* = −0.51, *P* < 0.05), chenodeoxycholic acid glycine conjugate (*r* = −0.57, *P* < 0.05), linoleic acid (*r* = −0.75, *P* < 0.001), arachidonic acid (*r* = −0.73, *P* < 0.001), and phosphorylcholine (*r* = −0.87, *P* < 0.001) and significantly positive associated with taurocholic acid (*r* = 0.49, *P* < 0.05).

### 3.8. Metabolic Pathway Analysis

**Figure 7:**
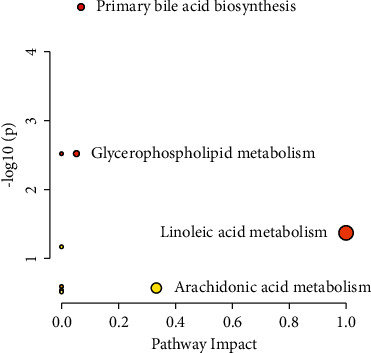
Metabolic pathway analysis based on potential markers identified in plasma metabolomics.

MetaboAnalyst was employed to elucidate the related pathways that were affected by WG-r on the hepatotoxicity. As shown in Figure 7, primary bile acid biosynthesis, glycerophospholipid metabolism, arachidonic acid metabolism, and linoleic acid metabolism were considered potential target pathways with high impact and low false discovery rate (FDR) (Table 3).

## 4. Discussion

Realgar has a good therapeutic effect on autoimmune diseases clinically [17]. Arsenic, the major active ingredient of realgar, has hepatotoxic effect, which greatly limits the clinical application of realgar [18]. Traditionally, in order to promote the absorption of realgar and enhance its curative effect, realgar is usually ground into very fine powder, which will increase its hepatotoxicity. Water-grinding realgar, a processed product by water, is usually used in clinic because of its lower toxicity compared with raw realgar. In this study, UPLC-HDMS-based on plasma metabolomics technique was applied to probe the alterations of metabolic profiles in the plasma of realgar-exposed rats and further revealed the hepatotoxicity mechanism of realgar via histopathology and biochemical assay. At the same time, the mechanism of realgar's hepatotoxicity reduction after water processing was expounded.

### 4.1. Biochemical Results and Histopathology

In our study, G-r was ground to the same particle size as WG-r, and they had many similar elements shown by elemental analysis, but the morphology of SEM analysis was different. The surface texture of WG-r was smooth and obtuse, with more fine powders attached to the surface, while G-r was more polyhedral with sharp edges. After rats were exposed to low (0.64 g/kg/day) and high (1.92 g/kg/day) doses of G-r and WG-r for one month, histopathologic examination was performed. There was no significant damage in the low-dose G-r group and WG-r group, compared with the control group. Hepatic histopathologic examinations showed that compared with the control group, in HG-r and HWG-r groups, cloudy swelling, cell cord derangement of hepatocytes, and hepatic sinusoid stenosis were observed. However, this pathological lesion returned to normal one week after WG-r withdrawal, but this recovery trend was not significant in Group CHG-r (Figure 2).

The hepatotoxicity of low-dose realgar is not obvious, and that the liver injury of high-dose realgar would be restored also appeared in the analysis of biochemical analysis. It is accepted that ALT and AST are critical biochemical indexes of evaluating liver function. Elevations of AST and/or ALT suggest hepatocellular injury, and ALT is a more specific marker of hepatic injury than AST [19]. Clinical biochemical parameters of serum showed that only high-dose realgar groups exhibited hepatotoxicity and the damage was reversible, which was indicated by a pronounced increase in serum ALT (Group HG-r, *P* < 0.05; Group HWG-r, *P* < 0.01) (Figure 3(b)) and a pronounced decrease in serum AST (Group HG-r, *P* < 0.01; Group HWG-r, *P* < 0.001) (Figure 3(c)). These significant differences were largely recovered after one week of discontinuation. SOD and GSH are physiological antioxidants against free radicals [20]. High-dose G-r and WG-r produce oxidative stress in the liver, as shown by the evidently decreased SOD activities and GSH contents and this injury might be recovered after drug withdrawal. These results indicated that G-r and WG-r at low doses may not be hepatotoxic, and the lesions elicited by high-dose realgar products might be recovered autonomously after withdrawal. Histopathology showed that the liver injury of G-r had no significant recovery trend. It can be inferred that the hepatotoxicity of G-r is greater than that of WG-r.

### 4.2. Biochemical Interpretation

The metabolic profile analysis of plasma samples not only supported the above findings but also further revealed the mechanism underlying hepatotoxicity of high-dose realgar. Using the metabolomics platform, we present evidence that rats exposed to high-dose realgar have a unique plasma endogenous metabolites signature. Bile acids (BAs) are natural components of bile, and they play an important role in absorption, excretion, and transportation of dietary lipids in the intestine and liver [21].

Primary bile acid is one of the most important pathways for the metabolism of cholesterol in mammals; the major cholic acid (CA) and chenodeoxycholic acid (CDCA) are produced from cholesterol by acidic, classical, or alternative pathways [22]. Glycocholic acid (GCA) is a secondary bile acid existing as a sodium salt in the mammalian bile, related to the emulsification of fats. As the final product of cholesterol, CDCA presumably inhibit the increase of serum triglyceride levels via inhibiting hepatic synthesis [23], and it is positively correlated with liver injury [24]. In our study, increased levels of CDCA, GCA, and CA were observed in the plasma of HG-r-exposed and HWG-r-exposed animals, which indicated that the primary bile acid biosynthesis showed severe perturbation after the treatment of high-dose realgar, which implied that elevated BA levels may be a consequence of liver injury and liver dysfunction.

Arachidonic acid (AA) is an integral constituent of cell membrane phospholipids, and it is plentiful in the liver, skeletal muscle, retina phospholipids, and brain [25]. In the ethanol-fed rats, the severity of liver pathology was inversely associated with the diminution in AA [26]. Linoleic acid (LA) is the most abundant and important essential fatty acid that cannot be synthesized de novo and exclusively extracted from diet [27, 28]. The evidence supported the idea that LA, easy to convert to AA [29], could inhibit hepatic lipogenesis, increase plasma oxylipin levels, and promote hepatic fatty acid oxidation. Increased levels of AA and LA in the plasma of high-dose-realgar-exposed rats were observed, which suggested that arachidonic acid metabolism and linoleic acid metabolism showed disturbance after the treatment of high-dose G-r and WG-r.

Lysophosphatidylcholine (LPC) could promote inflammation and is associated with the development of many diseases [30]. It could affect drug metabolism by enzymes that affect drug metabolism *in vivo*, aggravate metabolic disorders, and lead to liver diseases [31]. Our study found that the concentrations of LysoPC (16 : 0/0 : 0) and LysoPC (P-18 : 0/0 : 0) in rats decreased after oral administration of realgar. It could be seen that high-dose realgar caused the disorder of glycerophospholipid metabolism and might generate toxic metabolites, leading to the destruction of hepatocyte structure and apoptosis, as well as liver damage.

### 4.3. Relationship between Biochemical Parameters and Metabolite Differences

Pearson's correlation analysis for the potential biomarkers and biochemical indicators was generated to create a compendium metabolic profile that integrated the complementary information from the UPLC-HDMS and clinical chemistry analytical methods. As shown in Figure 6(b), cholic acid, chenodeoxycholic acid glycine conjugate, linoleic acid, arachidonic acid, and phosphorylcholine were found to be positively correlated to ALT but inversely correlated to AST. Linoleic acid and arachidonic acid were observed to be negatively correlated to SOD, MDA, and GSH. These correlations between biochemical parameters and potential biomarkers discovered in our study could offer a useful reference for understanding the pathological mechanisms how realgar induced hepatotoxicity.

### 4.4. Advantages and Limitations

The advantage of our study is to keep the particle size of G-r and WG-r at the same level, so as to avoid hepatotoxicity caused by different absorption rates due to the different particle sizes between G-r and WG-r. In this way, we can focus on the effect of the traditional processing method, which is called water grinding, rather than the particle size in realgar hepatotoxicity. Realgar processed with or without water made G-r and WG-r have the same particle size but different morphologies and different trace elements, resulting in different hepatotoxicity, which indicates that water grinding, a traditional Chinese medicine processing method, has the effect of attenuating toxicity. Limitations of this study should be noted. First, the difference of trace elements between G-r and WG-r had not been studied in this study, which might be the reason why the recovery of liver injury caused by WG-r was obvious, while the liver injury caused by G-r is more difficult to recover after one week's withdrawal. In addition, G-r and WG-r, which were two kinds of realgar processed by different processing methods, and their different mechanisms of hepatotoxicity need further study. Our laboratory has been carrying out research on these limitations.

## 5. Conclusion

UPLC-HDMS-based plasma metabolomics analysis combined with biochemical and histopathology assays was employed to investigate the metabolic profiles of the realgar's hepatotoxicity. Fourteen potential biomarkers were discovered, and these metabolites suggested that various pathways including the primary bile acid biosynthesis, arachidonic acid metabolism, linoleic acid metabolism, and glycerophospholipid metabolism. BAs, AA, LA, and LPCs were proposed as key metabolites related to disturbance induced by realgar. Water grinding is one of the main processing methods of traditional Chinese mineral medicine. It is believed that processed with water can reduce impurities, which is aimed to reduce side effects, and also make the particle size smaller to promote absorption, thereby increasing the efficacy. Our results demonstrated that G-r and WG-r had the same particle size and different processing methods. At the dose of 0.64 g/kg/day, the hepatotoxicity of G-r and WG-r was not obvious in rats. When the dose was increased to 1.92 g/kg/day, both G-r and WG-r showed significant liver damage. After one week's withdrawal, the liver damage caused by WG-r was significantly recovered, while the liver injury caused by G-r was more difficult to recover. This novel analytical method provides an ideal platform for elucidating the detoxification mechanism of traditional Chinese medicine processing.

## Figures and Tables

**Table 1:** The grouping of experimental animals.

Groups	Subgroups	Exposure
Control groups (*n* = 12)	Control group (Group C, *n* = 6)	0.5% (w/v) CMC-Na, 10 mL/kg/day, 30 days.
	Convalescent control group (Group CC, *n* = 6)	0.5% (w/v) CMC-Na, 10 mL/kg/day, 30 days; normal breeding, 7 days.

Grinding-realgar groups (*n* = 24)	Low-grinding-realgar group (Group LG-r, *n* = 6)	G-r, 0.64 g/kg/day, 30 days.
	Low-grinding-realgar convalescent group (group CLG-r, *n* = 6)	G-r, 0.64 g/kg/day, 30 days; normal breeding, 7 days.
	High-grinding-realgar group (Group HG-r, *n* = 6)	G-r, 1.92 g/kg/day, 30 days.
	High-grinding-realgar convalescent group (Group CHG-r, *n* = 6)	G-r, 1.92 g/kg/day, 30 days; normal breeding, 7 days.

Water-grinding-realgar groups (*n* = 24)	Low-water-grinding-realgar group (Group LWG-r, *n* = 6)	WG-r, 0.64 g/kg/day, 30 days.
	Low-water-grinding-realgar convalescent group (Group CLWG-r, *n* = 6)	WG-r, 0.64 g/kg/day, 30 days; normal breeding, 7 days.
	High-water-grinding-realgar group (Group HWG-r, *n* = 6)	WG-r, 1.92 g/kg/day, 30 days.
	High-water-grinding-realgar convalescent group (Group CHWG-r, *n* = 6)	WG-r, 1.92 g/kg/day, 30 days; normal breeding, 7 days.

**Table 2:** The identification of potential biomarkers in rats induced by realgar.

<!—Col Count:8<!—Metabolites	Formula	*t*_*R*_ (min)	Calculate (m/z)	Error (ppm)	ESI mode	HMDB ID	Related pathway
Taurocholic acid	C_26_H_45_NO_7_S	4.2	514.2822	4.3	—	HMDB0000036	Taurine and hypotaurine metabolism
Glycocholic acid	C_26_H_43_NO_6_	4.54	464.301	1.6	—	HMDB0000138	Primary bile acid biosynthesis
Chenodeoxycholic acid	C_24_H_40_O_4_	6.55	391.2843	2.8	—	HMDB0000518	Primary bile acid biosynthesis
Cholic acid	C_24_H_40_O_5_	5.42	389.2686	1.5	—	HMDB0000619	Primary bile acid biosynthesis
Chenodeoxycholic acid glycine conjugate	C_26_H_43_NO_5_	4.56	448.3062	1.4	—	HMDB0000637	Primary bile acid biosynthesis
Linoleic acid	C_18_H_32_O_2_	6.88	279.2325	1.6	—	HMDB0000673	Linoleic acid metabolism
Stearic acid	C_18_H_36_O_2_	10.89	283.2635	2.7	—	HMDB0000827	Biosynthesis of unsaturated fatty acids
Arachidonic acid	C_20_H_32_O_2_	6.92	303.2321	2.8	—	HMDB0001043	Arachidonic acid metabolism
Choline	C_5_H_14_NO	11.06	104.1066	9.0	+	HMDB0000097	Glycine, serine, and threonine metabolism
Phosphorylcholine	C_5_H_15_NO_4_P	6.3	184.0729	5.3	+	HMDB0001565	Glycine, serine, and threonine metabolism
5-Methoxyindoleacetate	C_11_H_11_NO_3_	2.9	188.0698	7.4	+	HMDB0004096	Glycerophospholipid metabolism
Palmitoyl L-carnitine	C_23_H_46_NO_4_	9.04	400.341	2.8	+	HMDB0000222	Fatty acid degradation
LysoPC (16 : 0/0 : 0)	C_24_H_50_NO_7_P	8.24	496.3371	5.4	+	HMDB0010382	Glycerophospholipid metabolism
LysoPC (P-18 : 0/0 : 0)	C_26_H_54_NO_6_P	9.42	508.3742	3.8	+	HMDB0013122	Glycerophospholipid metabolism

**Table 3:** Summary information of metabolic pathway analysis.

Pathway name	Match status	*P*	−log (*P*)	Holm *P*	FDR	Impact
Primary bile acid biosynthesis	5/46	2.27*E* − 05	4.6447	0.0019	0.0019	0.07
Biosynthesis of unsaturated fatty acids	3/36	0.003029	2.5188	0.25	0.084	0
Glycerophospholipid metabolism	3/36	0.003029	2.5188	0.25	0.084	0.05
Linoleic acid metabolism	1/5	0.04239	1.3727	1	0.89	1
Taurine and hypotaurine metabolism	1/8	0.06703	1.1737	1	1	0
Glycine, serine, and threonine metabolism	1/34	0.2573	0.5896	1	1	0
Arachidonic acid metabolism	1/36	0.27033	0.5681	1	1	0.33
Fatty acid degradation	1/39	0.2895	0.5384	1	1	0
Tryptophan metabolism	1/41	0.3020	0.5200	1	1	0

## Data Availability

All the data included in this article are available from the corresponding author upon request.
